# Differences in expression of Peroxisome Proliferator-activated Receptor-γ in early-onset preeclampsia and late-onset preeclampsia

**DOI:** 10.1186/s13104-020-05029-x

**Published:** 2020-03-27

**Authors:** W. Permadi, K. I. Mantilidewi, A. F. Khairani, U. A. Lantika, A. R. Ronosulistyo, H. Bayuaji

**Affiliations:** 1 Department of Obstetrics and Gynecology, Faculty of Medicine, Universitas Padjadjaran–Dr. Hasan Sadikin Hospital, Jl. Pasteur No. 38, Bandung, 40161 West Java Indonesia; 2 Division of Cell Biology, Department of Biomedical Sciences, Faculty of Medicine, Universitas Padjadjaran, Bandung, Indonesia; 3 Oncology and Stem Cell Working Group, Faculty of Medicine, Universitas Padjadjaran, Bandung, Indonesia; 4 Department of Histology and Medical Biology, Faculty of Medicine, Bandung Islamic University, Jl. Tamansari No.22, Bandung, 40116 West Java Indonesia

**Keywords:** PPAR-γ, Early onset preeclampsia, Late-onset preeclampsia, Trophoblast, Placenta

## Abstract

**Objective:**

PPARγ is a ligand-binding transcription factor that has been reported to be implicated in lipid metabolism, immune function, and cellular growth and differentiation. It has been suspected to play a role in the pathophysiology of preeclampsia, although the mechanism is yet to be elaborated. This study aims to investigate the expression of PPARγ in early onset preeclampsia (EOPE), late onset preeclampsia (LOPE), and normal pregnancy. We conducted this study using primary trophoblastic cell culture incubated with serum from EOPE, LOPE, and normal pregnancy. The expression of PPARγ in these cells was analyzed using Western Blot. Statistical analysis was performed using one-way ANOVA and Bonferroni’s post hoc test. p < 0.05 is considered significant.

**Results:**

Serum from normal pregnant women and EOPE did not induce any difference in the expression of PPAR-γ (p > 0.05). In contrast, expression of PPAR-γ was increased in those cells induced by serum from LOPE (p < 0.001). Therefore, we conclude that hypothetically PPAR-γ might play role in the pathophysiology of LOPE but not in EOPE. Other possibility is the activity of PPAR-γ in EOPE is inversely correlated with its expression, therefore the high enzymatic activity of PPAR-γ is tightly regulated by attenuating its expression.

## Introduction

Preeclampsia, a pregnancy-related hypertensive disorder, is among the most common complications in pregnancy. It is associated with a high maternal and perinatal mortality and morbidity [1, 2]. The pathogenesis of preeclampsia is still elusive, although several factors has been extensively well studied, such as defective placentation [3–6], ischemia of the uteroplacental circulation [5, 7–9], endothelial cell dysfunction [10, 11], and excessive inflammatory reactions to the invading trophoblast [12, 13].

Preeclampsia may be divided into two distinct entities, early- and late-onset. Early -onset preeclampsia (EOPE) occurs at < 34 weeks of gestational age, and late onset preeclampsia (LOPE), manifests at ≥ 34 weeks of pregnancy [14–16]. Both EOPE and LOPE are associated with different biochemical markers, genetic and environmental risk factors, prognosis, heritability, and clinical features. Evidence showed that several of the markers rose prior to pregnancies, and may persist or disappear before clinical manifestation of preeclampsia [17–19].

Peroxisome Proliferator-activated Receptor-γ (PPAR-γ) is a member of the nuclear receptor superfamily and has been reported to be implicated in key functions in the cells including cell proliferation and differentiation, inflammation and oxidation, glucose and lipid metabolism. These functions are important to ensure normal pregnancy development [20, 21]. In normal pregnancy, activators of PPAR-γ was increased along with gestational age. In a previous study that compared JEG-3 cell lines treated with serum extract either from women with normal pregnancy, mild, severe early-onset, or severe late-onset preeclampsia, Waite et al. demonstrated that PPARγ activators was reduced by 60% in the sera of severe early onset and by 55% in severe late onset preeclampsia compared to that of normal serum [22]. In contrast, Holdsworth-Carson et al. [23] demonstrated that placentas from women with pre-clampsia did not demonstrate any differences in mRNA or protein expression of PPAR-γ compared with healthy controls.

These studies above have suggested underlying differences in the biological mechanism of EOPE and LOPE. However, there are insufficient evidence to suggest how the expression of PPARγ may differ in EOPE and LOPE. Therefore, here we attempted to study the possible involvement of PPAR-γ in EOPE, LOPE compared to that of normal pregnancy.

## Main text

### Methods

#### Sample size selection

The lack of preceding studies made it difficult to conduct a priori power calculation. Therefore, our sample size was based on the work of Lazic [24], who stated that, for a cell culture study, an n of 3 patients per group is representative to see differences among each.

#### Cells

Our cellular model of preeclampsia, using primary trophoblastic cells derived from normal pregnancy incubated in sera from preeclamptic women was based on the work of Pramatirta et al. (dissertation, unpublished data), which in turn was based on the work of Neale et al. [25]. In our study, each cultured cell was meant to represent cellular models of normal, early-onset, and late-onset preeclampsia [25]. Cultured of primary trophoblastic cells obtained from term normal pregnancy was courtesy from Oncology and Stem Cell Working Group, Faculty of Medicine, Universitas Padjadjaran, Bandung, Indonesia. Cells were maintained in medium amniomax basal media (Gibco, USA) added with amniomax supplement (Gibco, USA), at 37 °C/5% CO_2_.

#### Maternal serum

Serum was courtesy from Oncology and Stem Cell Working Group, Faculty of Medicine, Universitas Padjadjaran, Bandung, Indonesia. Serum were obtained from normal pregnant women in the third trimester (n = 3), and from patients with EOPE (n = 3), and LOPE (n = 3). Pregnancies were considered normal when patients did not have medical and obstetric complications of pregnancy and delivered term, appropriate for gestational age neonate (≥ 37 weeks). Preeclampsia was defined as hypertension (systolic blood pressure ≥ 140 mmHg or diastolic blood pressure ≥ 90 mmHg on at least two occasions, 4 h to 1 week apart) and proteinuria (300 mg in a 24-h urine collection or one dipstick measurement of > 2+) [25, 26]. Patients with severe features, chronic hypertension, diabetes mellitus, systemic diseases such as antiphospholipid antibody syndrome, thrombophilia or transient blood pressure elevations were excluded from this study.

#### Exposure cells to serum

Cells were plated at 24-well and incubated for 24 h at 37 °C with 5% CO_2_. The cells were followed by treatment with serum from the patients at 15% final concentration [24–26] in amniomax (Gibco, USA) in the presence of amniomax supplement (Gibco, USA) for 24 h. The three groups were as follow: normal serum (normal), EOPE, and LOPE.

#### Whole cell lysate preparation and western blotting

Cells were washed with ice-cold 1 × PBS and then lysed in RIPA buffer in the presence of protease inhibitors and phosphatase inhibitors. Lysates were then added with SDS sample buffer (50 mM Tris–HCl pH 8.0, 150 mM NaCl, 1 mM EDTA, 1% SDS), and were centrifuged at 17,500×*g* for 20 min at room temperature. The resulting supernatants with equal amount of total protein was loaded in each lane. After transfer to PVDF, the membranes were blocked with 0.25% BSA in TBS-Tween for 30 min at room temperature. Primary and secondary antibody were incubated at 4 °C overnight, and 90 min at room temperature, respectively, with antibodies diluted in blocking buffer BSA 0.1%. Antibodies used included rabbit monoclonal antibody (mAb) against PPAR-γ (Cell Signaling Technology), mouse anti-actin mAb (Thermo Fisher Scientific), HRP-conjugated secondary antibodies against anti-rabbit, and anti-mouse was from Thermo Fisher Scientific and Santa Cruz, respectively. After treatment with ECL reagent (GE Healthcare), proteins in membranes were detected by C-Digit (Licor). Expression was quantified by densitometric scanning by Image-J followed by normalizing PPAR-γ expression to that of β-actin.

#### Statistical analysis

Data are presented as mean ± SEM from three separate experiments. Statistical analysis was performed by SPSS software, version 20.0 (SPSS Inc., Chicago), and p < 0.05 was considered significant (one-way ANOVA and Bonferroni’s test) versus serum normal pregnancy.

## Results

**Fig. 1:**
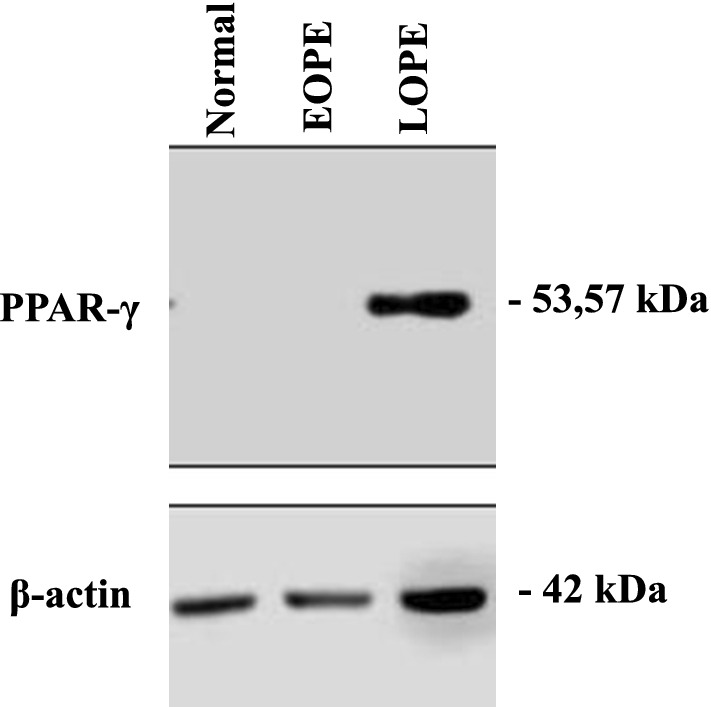
Expression of PPAR-γ. Primary trophoblastic cells were treated with normal serum (normal), EOPE, and LOPE. PPAR-γ proteins were subjected to immunoblot analysis with a mAb that recognizes PPAR-γ as well as with a mAb to β-actin (loading control)

The result of incubation of primary trophoblastic cells with serum from normal pregnancy, serum of EOPE, and LOPE, analyzed by Western Blot shown as follow in Fig. 1.

Our result showed the expression of PPAR-γ in primary trophoblastic cells treated by serum normal pregnancy compared to that of EOPE showed no expression in Western Blot. Remarkably, treatment of LOPE showed marked expression of PPAR-γ compared to that of normal pregnancy.

**Fig. 2:**
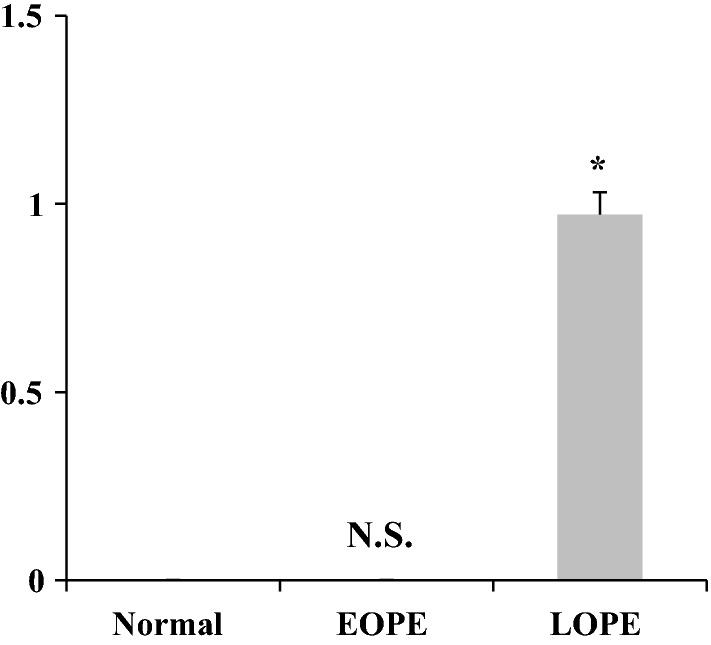
Quantification of Expression of PPAR-γ. Expression was quantified by densitometry scanning by Image-J followed by normalizing PPAR-γ expression to that of β-actin. Data are the mean ± S.E. from three separate experiments. versus serum normal pregnancy. *p < 0.01 (one-way ANOVA and Bonferroni’s test) versus normal pregnancy. *N.S.* non significant

Next, we confirmed the significance of our result. We performed quantification and showed the result in Fig. 2. The result was there was no difference in the expression of PPAR-γ in those cells treated by EOPE compared to normal pregnancy (p < 0.01), while the treatment of LOPE resulted in significant high expression of PPAR-γ compared to that of normal pregnancy (p < 0.001).

### Discussion

In our experiment, we used a primary trophoblastic cells obtained from normal pregnancy that has been exposed to either serum from normal pregnancy, EOPE, or LOPE [7, 27].

The majority of in vitro experiments were done as a prelude for translational research. Cell lines have limitations for preelampsia model due to the difficulties in interpretation compared to in vivo condition. Instead, cultured primary trophoblastic cells derived from isolated human trophoblasts were used as a model for preeclampsia [28]. This technique has been proven and used to model other placental functions, such as endocrinology, immunology, differentiation, and apoptosis in the placenta [28–30].

Previously, others have used cultured primary trophoblastic cells treated with serum of preeclamptic patients as models for preeclampsia. Pramatirta [29] found increased expression of TNF-α and caspase-3, and apoptotic index in preeclampsia serum-induced trophoblast cells compared to that of normal and controls. Other studies found structural derangement of vessels resembling disruption in interaction of trophoblastic cells with endothelial cells [26, 31]. In conclusion, treatment of primary trophoblastic cell with serum of preeclamptic patient is suitable as an in vitro model of the disease.

Peroxisome Proliferator-activated Receptor-γ (PPAR-γ) is a member of the nuclear receptor superfamily. They play major roles in diverse aspects of energy metabolism, inflammation, and development. Following ligand binding, PPARs form heterodimers with retinoid X receptors (RXRs), and bind to PPAR-response elements (PPREs) of target genes to activate transcription [32–34].

Among the three PPAR subtype identified, PPARγ has been reported to hold the most crucial role in placental development [20, 21]. Homozygous PPAR-γ^−/−^ mice embryos died due to severe placental dysfunction [35, 36]. In human pregnancy, PPARγ regulates proinflammatory mediators such as IL-6, IL-8, TNF-α, as well as trophoblastic function, and disruption to these functions may result in severe gestational disorders [20, 36]. PPAR-γ antagonists used in in vitro study of first trimester extravillous trophoblasts resulted in increased trophoblastic invasion, while agonist inhibited it. These data support the role of PPAR-γ in the regulation of trophoblasts invasion to decidua [21, 36].

Previous studies have found that PPAR-γ was expressed in human placenta from the beginning to the end of pregnancy [37–39]. This same research strengthened the possible involvement of PPAR-γ in normal pregnancy because they found PPAR-γ expression was preserved until third trimester [40].

In our study, treatment of the primary trophoblastic cells with serum from LOPE induced high expression of PPAR-γ. By contrast, treatment using EOPE and normal pregnancy serum did not induce PPARγ expression. These results strongly imply the possible involvement of PPARγ in preeclampsia. Handschuh et al. [41] stated PPAR-γ activity depends on the trophoblast subpopulation, gestational age, and types of stimulating ligands. Therefore, different content of serum in EOPE and LOPE has different effect to PPAR-γ expression.

In LOPE, maternal factors such as obesity, hyperlipidemia, diabetes andchronic hypertension highly contribute to the emergence of the disease [42–45] In our experiment the high expression of PPAR-γ induced by treatment with serum of LOPE may indicate PPAR-γ expression is inversely correlates with its activity. As Levytska et al. [46] showed, agonist PPAR-γ rosiglitazone induced reduction in the receptor expression in the BeWo cell line, an established model of synctitiotrophoblast formation in vitro, as well as in primary trophoblast cells. In contrast, inhibition of PPAR-γ activity by T0070907 caused extreme enhancement of receptor expression.

The evidence stated above suggests the activity of PPAR-γ is modulated by negative feedback [46]. Similarly, Levytska et al. [46] and Knabl et al. [47] stated PPAR-γ which act as as transcription factor plays important roles in fat and glucose metabolism, as well as cell growth and differentiation, so that tight autoregulation is necessary to even out its activity. A high activity will induce lower expression, and vice versa.

The two-stage theory of preeclampsia explained EOPE as such: first, defective trophoblastic invasion results in shallow placentation and impaired remodeling of muscular layer in spiral arteries. This process leads to the inability of the spiral artery to fully dilate and support a normal pregnancy. In the second stage, failure to establish adequate uteroplacental blood flow leads to relative trophoblastic hypoxia, eliciting an oxidative stress response which initiates the release of placental debris in maternal circulation and emerged as symptomatic disease. As a consequence for abnormal trophoblastic invasion at early placental development will result in earlier disease manifestation (< 34 weeks) [42–45] In EOPE, this abnormal trophoblastic invasion may be regulated by PPAR-γ, however high activity of this receptor result in diminished receptor expression [46, 47].

### Conclusion

PPAR-γ might play role in the pathophysiology of LOPE but not in EOPE. Other possibility is the activity of PPAR-γ in EOPE is inversely correlated with the expression, therefore the high enzymatic activity of PPAR-γ is tightly regulated by attenuating its expression.

## Limitation of the study

This study is limited by a possible discrepancy among gene expression, protein expression, and protein activity. Gene activities describe transcription process at mRNA level, while protein expression is highly modified at translational process or post translation, as well as protein degradation rate. Protein activity may be affected by chemical reaction, therefore may influence by presence of catalisator or inhibitor. Further research must confirm the mechanism of higher expression of PPAR-γ in our research at pre- or post-receptor level [48].

Secondly, a very small sample size carries a high risk of type 1 error. Thirdly, we were unable to evaluate other active serum constituents found in the sera of preeclamptic women, such as sFlt-1 and PlGF, and investigate whether they are directly involved in the PPARγ pathway [49]. Further research could be conducted to investigate the direct effect of PPARγ by treating the sample with PPARγ-antibody or specific activator or inhibitor prior to serum addition, or by eliminating other active constituents prior to treatment with the serum.

## Data Availability

Availability of data and materials are available upon request to the corresponding author. The cells and serum used as archived biological materials are courtesy from Oncology and Stem Cell Working Group, Faculty of Medicine, Universitas Padjadjaran, Bandung, Indonesia.

## Abbreviations

EOPE: Early-onset preeclampsia
LOPE: Late-onset preeclampsia
PPAR-γ: Peroxisome Proliferator-activated Receptor-γ
PPRE: PPAR-response elements
RXR: Retinoid X receptors
